# Bone mineral density in patients with multiple sclerosis, hereditary ataxia or hereditary spastic paraplegia after at least 10 years of disease - a case control study

**DOI:** 10.1186/s12883-016-0771-4

**Published:** 2016-12-05

**Authors:** Cecilia Smith Simonsen, Elisabeth Gulowsen Celius, Cathrine Brunborg, Chantal Tallaksen, Erik Fink Eriksen, Trygve Holmøy, Stine Marit Moen

**Affiliations:** 1 Department of Neurology, Drammen Hospital, Vestre Viken HF, Dronnigsgate 28, 3004 Drammen, Norway; 2 Department of Neurology, Oslo University Hospital, Oslo, Norway; 3 Department of Neurology, Akershus University Hospitals, Oslo, Norway; 4 Department of Endocrinology, Morbid Obesity and Preventive Medicine, Oslo University Hospital, Oslo, Norway; 5 Institute of Clinical Medicine, Faculty of Medicine, University of Oslo, Oslo, Norway; 6 Institute of Health and Society, Faculty of Medicine, University of Oslo, Oslo, Norway; 7 Oslo Centre for Biostatistics and Epidemiology, Research Support Services, Oslo University Hospital, Oslo, Norway

**Keywords:** Bone Mineral Density, Case control, Hereditary Ataxia, Hereditary Spastic Paraparesis, Multiple Sclerosis, Osteoporosis

## Abstract

**Background:**

Although disability is considered the main cause of low bone mineral density (BMD) in multiple sclerosis (MS), other factors related to the disease process or treatment could also be involved. The aim of this study was to assess whether patients with MS are more likely to develop low BMD (osteopenia or osteoporosis) than patients with the non-inflammatory neurological diseases Hereditary Spastic Paraplegia (HSP) and Hereditary Ataxia (HA).

**Methods:**

We performed a case control study comparing BMD (spine, hip and total body) and biochemical measures of bone metabolism in 91 MS patients and 77 patients with HSP or HA, matched for age, gender and disability. Both patient groups had lived with the disease for at least 10 years.

**Results:**

In total 74.7% of the patients with MS and 75.3% of the patients with HSP or HA had osteopenia *(−2.5 < T- score < −1.0)* or osteoporosis *(T- score ≤ −2.5)* in one or more sites. Osteoporosis was more common in patients with MS than with HSP/HA (44.0 vs 20.8%, *p* =0.001). This difference was not significant after correction for confounders (*p* = 0.07), nor were any of the biochemical markers.

**Conclusion:**

Most patients with disabling neurological diseases like MS and HSP/HA develop osteopenia or osteoporosis. MS patients had osteoporosis more frequently than HA/HSP patients, though the difference was not significant after adjusting for confounders. Osteoporosis and bone health should be considered in all patients with both inflammatory and degenerative chronic neurological diseases.

**Electronic supplementary material:**

The online version of this article (doi:10.1186/s12883-016-0771-4) contains supplementary material, which is available to authorized users.

## Background

Multiple sclerosis is a chronic demyelinating disease of the central nervous system with a lifelong disease course and increasing physical disability. Patients with long-standing MS also have an increased risk of osteoporosis and fractures due to reduced bone mass and falls [1]. We have earlier found that low bone mass was more prevalent in newly diagnosed patients with MS with no or minor physical disability than in healthy controls [2]. Disability leading to disuse and reduced mechanical loading of bone is likely an important cause of osteoporosis in patients with long-standing MS [3]. Whether other factors contribute to reduced bone mineral density (BMD) is less clear [4, 5], though these may include etiologic or pathophysiologic factors shared between MS and osteoporosis. The skeleton harbours the bone marrow where immune competent cells and osteoclasts develop. The inflammatory processes of MS could possibly affect bone homeostasis as several cytokines, receptors, signalling molecules and transcription factors seem to be involved both in the pathogenesis of MS and in the differentiation and activation of osteoclasts [2, 6]. Moreover, hypovitaminosis D is detrimental for bone health and is also considered a risk factor for MS [7]. Different treatments may also contribute to bone loss in MS. Acute MS exacerbations are treated with glucocorticoids, a medication generally known to cause osteoporosis. Transient glucocorticoid pulses in MS have been reported to have no long-term adverse bone effects [8, 9], but the role of steroid treatment over time is not settled [5].

Hereditary ataxia (HA) and hereditary spastic paraplegia (HSP) are neurodegenerative disorders, which are thought to have little or no inflammation. HA is characterised by progressive gait and limb ataxia, loss of coordination and disturbances of speech and oculomotor control, whereas HSP is mainly characterized by progressive spasticity and weakness in the lower limbs [10]. There are very few studies focusing on bone health in HSP/HA patients. One small study on Friedreichs ataxia and a case control study on patients with spinocerebellar degeneration (both subtypes of HA) found significantly reduced bone mineral density compared to healthy controls [11, 12]. HA and HSP affect locomotion to the same extent as MS, but are considered primarily neurodegenerative and are not treated with glucocorticoids. These diseases are therefore relevant for charting the possible impact of inflammation and treatment on bone health in MS. The aim of this study was to compare the occurrence of osteoporosis and osteopenia, and also biochemical parameters of bone metabolism, in patients with HA and HSP with that of MS.

## Methods

### Study design and participants

Due to limited numbers of patients with the non-inflammatory neurological diseases HA and HSP, we first recruited 77 patients with HA and HSP identified from a registry at Oslo University Hospital and living within accessible distance from Oslo [10]. We subsequently recruited 91 matched patients with MS according to McDonald [13] or Poser [14] criteria with disease duration over 10 years from the MS clinics at Oslo University Hospital and Akershus University Hospital for this regional case control study. The MS patients were matched to HSP/HA patients by age (±5 years), gender and level of disability based on the Expanded Disability Status Scale (EDSS) [15]. Only patients that were mobile enough to get on and off the DXA machine bench with minimal assistance were included.

### Data collection

All BMD measurements were done between January and October in 2008. BMD was assessed from DXA-measurements of lumbar spine (anterior-posterior, L1-L4), left hip (femoral neck, femoral trochanter and total hip) and total body were performed with the same instrument at Aker Hospital in Oslo (Lunar Prodigy, General Electrics) and analysed using manufacturer specifications and normative data (National Health and Nutrition Examination Survey III population). The machine was calibrated daily according to manufacturer specifications, and control scanning with a phantom was used regularly to avoid drifting of the DXA measurements. Laboratory personnel handled the patients as a part of routine examination and did not have responsibility for analysis of data. The results are expressed as BMD (g/cm2) and T-score (the number of SDs by which a given BMD value differs from the mean reference value for healthy, young adults) (http://www.uptodate.com/contents/bone-density-testing-beyond-the-basics). We used the WHO classification of osteopenia (−2.5 < T-score < −1.0) and osteoporosis (T-score ≤ −2.5) [16]. Anthropometric data was measured simultaneously. Body mass index (BMI) ≥25 was classified as overweight (http://www.who.int/topics/obesity/en/)-linked. All biochemical measurements were performed as previously described [17]. Briefly, 25(OH)D and 1,25(OH)_*2*_D and DBP were measured by radioimmunoassay. Parathyroid hormone (PTH) was measured with non-competitive immunoluminometric assay. Serum bALP was measured by an enzyme immunoassay kit. Serum ionized calcium (iCa), creatinine, and phosphate were measured according to standard laboratory techniques. NTX in the second morning void urine was measured by competitive enzyme immunoassay. All measurements were collected at the same time as the DXA was performed.

All participants filled in a questionnaire concerning previous and current medication, other autoimmune diseases, sun exposure, exercise, dietary supplements, alcohol and smoking. The questionnaires included questions used routinely as a supplement to the DXA scan to register possible confounders with skeletal effects and were returned by mail. A neurologist recorded clinical history and EDSS based on the last clinical assessment in the case files and a phone call immediately prior to the DXA appointment. Self-reported data on medication were compared with hospital files for the patients with MS from Oslo University Hospital. We also calculated an EDSS equivalent for the HSP/HA patients based on the same criteria as MS patients.

### Statistical analysis

Results are expressed as mean ± standard deviation (SD) or proportion unless otherwise stated. Pearsons *X* ^2^ test for contingency tables was obtained to detect associations between categorical variables. Differences in continuous variables were tested with the independent sample *t*-test. Pearsons correlation coefficient (r) was used to analyse correlations between continuous variables. Only variables with significant relationship with both the MS patients versus HSP/HA patients as exposure and BMD (or T-score) as outcome were considered as possible confounders. Adjustments for multiple confounding variables were performed using multivariable linear regression. The possible confounders were analysed as independent variables separately and simultaneously in the regression models. The following outcome variables were analysed as dependent: L1-L4, left femoral total hip, neck and trochanter, total body, PTH, and phosphate. Missing values were not replaced. All statistical analyses were performed using IBM SPSS statistics version 21 (IBM SPSS Inc., Chicago, IL), and findings were considered significant if *p* < 0.05.

## Results

**Fig. 1:**
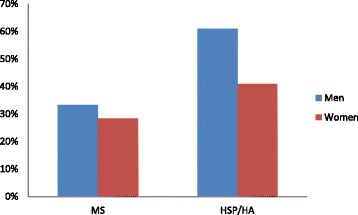
Percentage of MS patients and HSP/HA patients s with BMI (Body Mass Index) ≥ 25 (overweight or obese) by gender

The patient characteristics are shown in Table 1. The MS patients were grouped by disease course. There are patients with relapsing remitting MS (RRMS) that are still experiencing relapses, secondary progressive MS (SPMS), which now only have progressive disease and no relapses, and primary progressive MS (PPMS), which have had progressive disease from the start. The mean EDSS was 4.9 (median 5.0, range 6.5) in the MS group and when applying the same definitions of the expanded disability status score to the HA/HSP group, EDSS was 5.3 (median 6.0, range 5.0). The HA/HSP group had higher mean BMI (β_unadj._ = 1.93, *p* = 0.004) and body weight (β_unadj_ = 5.38, *p* = 0.03) compared to the MS group. This was particularly pronounced among the men where 61% of HA/HSP patients and 33.3% of MS patients were overweight or obese (*p* = 0.01) See Fig. 1.

**Table 1:** Demographic and anthropometric data on all patients, MS patients and HSP/HA patients

	All	MS	HSP/HA
Number (%)	168 (100)	91 (54.4)	77 (45.6)
Women (%)	82 (48.8)	46 (50.5)	36 (46.8)
Disease phenotype (%)		RRMS 51 (56.0) SPMS 34 (37.4) PPMS 6 (6.6)	HSP 61 (79.2) HA 12 (15.6) HSP w/ataxia 4 (5.2)
Age (SD)	55.3 (±10.4)	52.0 (±10.3)	52.7 (±10.6)
Disease duration >20 years (%)	93 (55.4)	46 (50.5)^a^	47(61.0)
Years since onset (SD)	24.2 (±12.8)	21.3 (±9.0)^a^	27.7 (±15.4)
Disability (SD)			
No aid	84 (50.0)	49 (53.8)	35 (45.5)
One or two sticks	65 (38.7)	31 (34.1)	34 (44.2)
In wheel chair	19 (11.3)	11 (12.1)	8 (10.4)
EDSS		4.9 (median 5.0, range: 1.5-8.0)	
Height, cm (SD)	172.7 (±8.6)	173.2 (±8.3)	172.1 (±8.9)
Weight, kg (SD)	74.3 (±16.3)	71.8 (±14.1)^a^	77.2 (±18.2)
BMI Kg/m^2^ (SD)	24.8 (±4.5)	23.9 (±4.2)^a^	25.8 (±4.7)
Total body fat % (SD)	32.3 (±8.5)	31.6 (±8.5)	33.0 (±8.5)

*Abbreviations: EDSS* Expanded Disability Status Scale*, RRMS* relapsing remitting multiple sclerosis*, SPMS* secondary progressive multiple sclerosis*, PPMS* primary progressive multiple sclerosis*, HSP* hereditary spastic paraplegia*, HA* hereditary ataxia*, BMI* body mass index

^a^ *p* <0.05 (independent sample *t*-test) compared to patients with HSP/HA

### BMD and T-scores

**Fig. 2:**
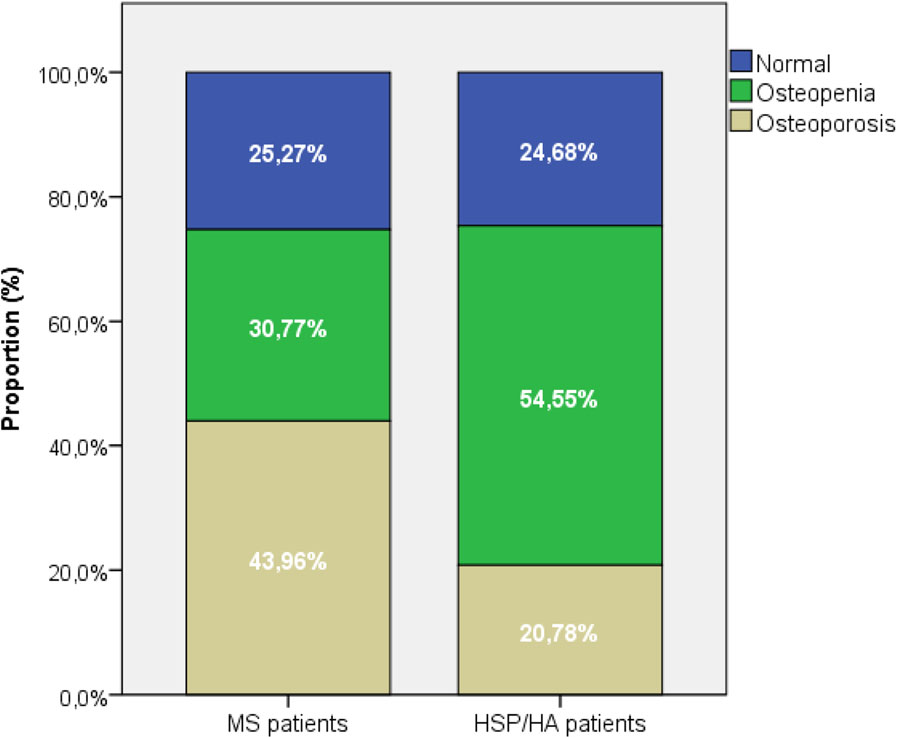
Proportion of patients with osteopenia (−2.5 < T- score < −1.0) and osteoporosis (T-score ≤ −2.5) in at least one site compared to HSP/HA patients

Unadjusted BMD and T-scores at each skeletal site are shown in Table 2. There was a tendency towards lower BMD and T-scores in the spine of patients with MS compared to patients with HA/HSP. The proportion of MS and HSP/HA patients with osteopenia or osteoporosis in at least one skeletal site is shown in Fig. 2. About 75% in each group exhibited osteopenia or osteoporosis. However, 44.0% of MS patients had osteoporosis in at least one site compared to 20.8% of HSP/HA patients (*p* = 0.001).

**Table 2:** BMD and T-scores. Four patients (2 with MS and 2 with HSP/HA) had hip replacements in the left hip and DXA was performed on the right side. Three patients did not have DXA measurements of either hip (1 MS patient with bilateral hip implants and 2 HSP/HA patient with either bilateral hip implants or implant in right hip and rotation osteotomia of the left hip), thus findings in hips only include 90 MS patients and 75 controls

Measurement	MS (*n* = 91)	HSP/HA (*n* = 77)
L1-L4		
BMD, g/cm^2^	1.07 ± 0.18	1.13 ± 0.16
T-score	−1.09 ± 1.51	−0.59 ± 1.31
L femoral total hip		
BMD, g/cm^2^	0.86 ± 0.17	0,90 ± 0.16
T-score	−1.49 ± 1.40	−1.17 ± 1.22
L femoral neck		
BMD, g/cm^2^	0.84 ± 0.15	0.87 ± 0.15
T-score	−1.43 ± 1.26	−1.22 ± 1.21
L femoral trochanter		
BMD, g/cm^2^	0.69 ± 0.16	0.73 ± 0.16
T-score	−1.56 ± 1.45	−1.20 ± 1.30
Total body		
BMD, g/cm^2^	1.12 ± 0.11	1.15 ± 1.12
T-score	−0.6 ± 1.40	−0.31 ± 1.33

*Abbreviations: BMD* bone mineral density

The difference between MS and HSP/HA patients’ lumbar spine BMD were significant, but not after adjusting for confounders (Additional file 1: Table S1), specifically BMI (Table 3). After controlling for the confounding effects of BMI and fish oil intake, the higher percentage of MS patients with osteoporosis compared to HSP/HA patients was no longer significant (*p* = 0.07).

**Table 3:** Differences in T-scores between MS patients and HSP/HA patients, with and without adjustment for confounders using linear regression analysis

	Unadjusted			Adjusted^a^		
Measurement	B	95% CI for β	*p* value	B	95% CI for β	*p* value
L1-L4	0.51	0.07, 0.94	0.02	0.32	−0.17, 0.80	0.20
L femoral total hip	0.32	−0.09, 0.76	0.13	0.15	−0.28, 0.58	0.50
L femoral neck	0.22	−0.16, 0.60	0.26	0.12	−0.90, 0.525	0.58
L femoral trochanter	0.36	−0.06, 0.79	0.10	0.22	−0.24, 0.68	0.35
Total body	0.29	−0.13, 0.71	0.17	0.07	−0.37, 0.51	0.76

*Abreviations: β* unstandardised regression coefficient

^a^Adjusted for BMI (body mass index) and fish oil intake

### Biochemical markers of bone metabolism

MS patients exhibited significantly lower PTH (β_unadj._ = 1.0, *p* = 0.002) and significantly higher phosphate (β_unadj._ = 0.07, *p* = 0.009) compared to HSP/HA. However, after adjustment for the confounding effect of corticosteroid use, disease duration, BMI and alcohol use, the difference in PTH and phosphate was no longer significant (Table 4).

**Table 4:** Comparing biochemical measures in MS patients and control patients (HSP/HA) with *P*-values, both unadjusted and adjusted

	MS	HSP/HA	Unadjusted β (95%CI)	*P*-value	Adjusted β (95%CI)	*P*-value
25(OH)D	77.5 ± 28.9	70 ± 26.2	−7.5 (15.9, 1.0)	0.08	−7.5 (15.9, 1.0)	0.08
1,25(OH) _*2*_D	139.8 ± 55.9	129.0 ± 36.2	−10.8 (−25.4, 3.9)	0.2	−10.8 (−25.4, 3.9)	0.2
PTH	3.7 ± 2.0	4,7 ± 2.0	1.0 (0.4,1.6)	0.002	0.17 (−0.6,1.0)	0.7*
iCa	1.3 ± 0.1	1.3 ± 0.1	0.0 (−0.01, 0.01)	0.8	0.0 (−0.01, 0.01)	0.8
Phosphate	1.1 ± 0.2	1.0 ± 0.2	−0.1 (−1.2, 0.02)	0.009	0.0 (−0.1, 0.1)	0.8**
DBP	4.2 ± 0.6	4.3 ± 0.8	0.1 (−0.1, 0.3)	0.5	0.1 (−0.1, 0.3)	0.5
Creat	67.7 ± 12.8	70.6 ± 14.4	2.9 (−1.3, 7.0)	0.2	2.9 (−1.3, 7.0)	0.2
bALP	23.4 ± 8.2	25.0 ± 6.1	1.6 (−0.6, 3.8)	0.2	1.6 (−0.6, 3.8)	0.2
NTX	46.8 ± 20.9	54.1 ± 36.3	7.3 (−1.7, 16.2)	0.1	7.3 (−1.7, 16.2)	0.1
Total Ca	2.4 ± 0.1	2.4 ± 0.1	0.0 (−0.01, 0.01)	0.9	0.0 (−0.01, 0.01)	0.9
Albumin	44.9 ± 2.4	45.3 ± 2.5	0.4 (−0.4, 1.1)	0.3	0.4 (−0.4, 1.1)	0.3
TSH	1.3 ± 0.7	1.3 ± 0.6	0.0 (−0.2, 0.2)	0.9	−0.0 (−0.2, 0.2)	0.9

Some variables have missing values due to technical errors (number of missing patients in parentheses): iCalcium (2), total Ca (2), phosphate (3), creatinin (3), albumin (3), thyroid stimulating hormone (TSH) and NTX (2) from MS patients and PTH (1), iCa (1), total Ca (1), phosphate (2), albumin (1) and NTX (2) from HA/HSP patients

*Abbreviations: 25(OH)D* 25-hydroxyvitamin D*, 1,25(OH)*_*2*_ *D* 1,25-dihydroxyvitamin D (active vitamin D metabolite)*, PTH* parathyroid hormone*, iCa* ionized calcium, *DBP* vitamin D binding protein*, bALP* bone specific alkaline phosphatase, *NTX* cross linked N-terminal telopeptide of type 1 collagen, *Ca* calcium*, TSH* thyroid stimulating hormone

*Adjusted for corticosteroid use, disease duration and alcohol use

**Adjusted for corticosteroid and BMI

## Discussion

The main finding in this study was that low bone mass was prevalent in both MS patients and HSP/HA patients, with osteoporosis being twice as common in the MS group. There were however no significant differences between the two patient groups after adjusting for relevant confounders.

Our study focused on the bone mass of patients with long-standing disease, but still mobile enough to get on a bench with minimal assistance. A broad approach by including measurements of BMD, biochemical parameters and registering possible confounders with skeletal effects was used to address possible factors involved in the bone loss in these patients in the course of disease.

BMD is influenced by a number of factors, including gender, age, ethnicity, BMI, smoking, physical exercise, vitamin D and certain drugs [18]. Our study highlights the importance of checking and adjusting for confounders when studying bone health in MS. There is a well-known association between body mass index (BMI) and bone mineral density [19–21] and although numerous studies have proven that MS patients have lower bone mineral density compared to HSP/HA patients [22–24], not all studies have adjusted for body weight or body size in the analysis [25, 26]. According to the Norwegian institute of public health (http://www.fhi.no/eway/default.aspx?pid=239&trg=Content_6496&Main_6157=6263:0:25,6306&MainContent_6263=6496:0:25,6313&Content_6496=6178:54371:25,6313:0:6562:16:::0:0) 2/3 of Norwegian 40–42 year olds were overweight or obese (65% of men and 44% of women). HSP/HA patients were thus representative (61% of HSP/HA men and 41% of HSP/HA women were overweight or obese). The MS patients on the other hand were slimmer as only 33.3% of men and 28.3% of women had BMI > 25. We know that a high BMI in childhood and adolescence is associated with a higher risk of MS [27, 28], but this is less obvious in patients with established MS [29]. The fact that patients with MS were slimmer than those with HSP and HA could reflect underlying differences in the pathogenesis of the diseases.

The biochemical markers of bone turnover and metabolism did not differ significantly between these MS patients and HA/HSP patients with over 10 years of disease. It is worth noting that these blood tests represent a snap shot of the dynamics of current bone metabolism, and do not, as BMD, reflect prior bone metabolism over time. However, the bone deficit found in our patients is unlikely to be due to recent increases in bone turnover.

Both HSP/HA patients and MS patients had a long disease history with mean disease duration exceeding 20 years. One limiting factor in MS and HSP/HA patient selection was that only patients who were mobile enough to independently get on and off the bench of the DXA machine were included. This entailed a selection towards the more mobile MS, HA and HSP patients. 50.0% did not use any walking aid (53.8% in the MS group and 45.5% in the HSP/HA group), a fairly low number in patients with disease duration above 20 years [30]. EDSS was 4.9 in the MS group and equivalent to 5.3 in the HSP/HA patients. Although we did not examine all the patients ourselves as we relied on recent clinical examinations noted in the patient case journal and additional phone calls to the patients prior to DXA appointment, we believe that this dual approach gave reliable data on EDSS and disability. Studies have shown that scoring EDSS can be done reliably by telephone, especially in patients with a higher EDSS, like our patients [31].

More MS patients had used corticosteroids compared to patients with HSP/HA, but only 3 MS patients reported more than five pulsed steroid courses during their life-time. The amount of steroids used is difficult to assess exactly retrospectively. We had to rely on the patients reporting steroid treatment in the questionnaire, which is subject to recall bias. These self-reported data were compared with hospital files where possible to increase the validity. The vast majority of our patients had not been exposed to large amounts of corticosteroids, and as expected from previous studies on the effect of steroid pulses on BMD in MS [8, 9], steroid use was not associated with BMD. Thus, steroid use is unlikely to be the cause of low bone mass.

We have previously shown that low bone mass was more prevalent in patients with newly diagnosed MS and clinically isolated syndrome (CIS) with no or minor physical disability compared to healthy controls [2] suggesting that etiological factors such as hypovitaminosis D affect BMD or that MS may affect bone haemostasis through inflammatory activity. It is a limitation of our study that we did not measure inflammatory markers, which may reflect disease activity in MS. As for vitamin D, these would however provide limited information about previous levels that is likely more relevant for BMD. However, if inflammation was a major driver of bone loss in MS, we would expect MS patients to have lower BMD compared to disability matched HSP/HA patients as HSP/HA is a non-inflammatory neurodegenerative disease with similar neurological symptoms. One explanation could be that the majority of our MS patients had developed a disease driven primarily by degeneration and less by inflammation. Some claim that during the later stages of the disease the remaining inflammation becomes trapped behind a closed or repaired blood–brain barrier and is not derived peripherally [32]. Our current results may indirectly suggest that the systemic inflammation in MS is not sufficient to increase bone loss over time as is seen in other diseases in which systemic inflammation dominates, such as inflammatory bowel disease [33]. Another possible explanation for the lack of significant difference between MS and HSP/HA patients could be that inflammation may also play a role in HSP/HA. The role of cytokines and microglia in spinocerebellar ataxias is just beginning to be investigated, with promising perspectives [34–36]. The role of inflammation in hereditary neurodegenerative disorders warrants more studies [37].

Although inflammation may be an integral part of bone loss in early MS, this may be only one of many factors behind bone depletion as the patient becomes more disabled. The findings of reduced BMD in MS patients are similar to that found in patients with other non-inflammatory, neurological disorder including stroke [38, 39]. As the level of disability in both MS patients and HSP/HA patients was fairly equal in our study, immobility is likely the main reason for high levels of osteoporosis and osteopenia in both groups.

Regardless of the mechanism, both patient groups have a high rate of osteoporosis and osteopenia and patients with MS had higher prevalence of osteoporosis than those with HSP/HA. According to the WHO, 15% of all Caucasians between 50 and 59 have osteoporosis [39–41] (http://www.who.int/chp/topics/rheumatic/en/) [40]. One study from Oslo found that 14–36% of women older than 50 years of age had osteoporosis [41]. In our study 33% of all patients (MS and HSP/HA patients) had osteoporosis and their age range was 24 to 79 (mean 52). If only looking at female patients aged 50 or older, (*n* = 46), this number rose to 52.2%. Osteoporosis is considered a major risk factor for fractures. One Danish registry study found an incidence rate of any fracture yielded 22.8 per 1000 person-years [42], and several studies confirm the increased risk of fractures in MS patients [1, 43, 44]. The patients are generally less mobile, have poorer balance and a high risk of falling [45] compared to healthy controls. One study found that more than 50% of 700 MS patients aged 55 years or older reported injurious falls, 12% within the last 6 months [46]. Patients with HSP are also at an increased risk of falls [47]. There are few studies on fracture risks in this patient group, though one case control study found that 24% of patients with spinocerebellar degeneration had at least one fracture over a 10 year period compared to 3% in healthy controls [12]. Regardless of cause, osteoporosis is a big public health problem. Fractures are associated with significantly reduced quality of life through pain, suffering and disability while hip fractures can cause death [48]. Our study highlights the importance of considering bone health in all patients with chronic, disabling neurological disease.

## Conclusion

Most patients with disabling neurological diseases develop osteopenia or osteoporosis. We did a case control study comparing bone mineral density and biochemical measures of bone metabolism in MS patients and patients with HSP or HA, matched for age, gender and disability. MS patients had osteoporosis more frequently than HA/HSP patients, though the difference was not significant after adjusting for confounders. Osteoporosis and bone health should be considered in all patients with both inflammatory and degenerative chronic neurological diseases.

## Additional file

Additional file 1: Supplementary Table 1: an overview of possible bone influential factors in both groups. (DOCX 14 kb)

## Abbreviations

1.25(OH)2D: 1,25-dihydoxyvitamin D (active vitamin D metabolite)
25(OH)D: 25-hydroxyvitamin D
B: Unstandardised regression coefficient
bALP: Bone specific alkaline phosphatase
BMD: Bone mineral density
BMI: Body mass index
Ca: Calcium
CIS: Clinically isolated syndrome
DBP: Vitamin D binding protein
DXA: Dual-energy X-ray absorptiometry
EDSS: Expanded disability status scale
HA: Hereditary ataxi
HSP: Hereditary spastic paraplegia
iCa: Ionized calcium
MS: Multiple sclerosis
NTX: Cross linked N-terminal telopeptide of type 1 collagen
PTH: Parathyroid hormone
SCA: Spinocerebellar ataxia
SD: Standard deviation
TSH: Thyroid stimulating hormone
WHO: World health organisation
