# Supplementary material for: Bone mineral density in patients with multiple sclerosis, hereditary ataxia or hereditary spastic paraplegia after at least 10 years of disease - a case control study
Source: BMC Neurol. 2016 Dec 5;16:252. doi: 10.1186/s12883-016-0771-4 (PMC5139093; doi:10.1186/s12883-016-0771-4)
Supplement: Additional file 1: — Supplementary Table 1: an overview of possible bone influential factors in both groups. (DOCX 14 kb) [file 12883_2016_771_MOESM1_ESM.docx]

| **Possible bone influential factors** | **MS patients** | **HSP/HA patients** |
| --- | --- | --- |
| Ever used iv or PO Corticosteroids (%)† | 59 (68.6) | 8 (11.9) |
| Other relevant medications† (%) | 30 (34.9) | 11 (15.7) |
| Other relevant diseases (%) | 15 (17.6) | 9 (12.9) |
| Smoker (%) | 28 (33.3) | 19 (26.8) |
| Alcohol >1/week (%) | 32 (35.2) | 14 (18.2) |
| Takes fish oil (%)†* | 75 (82.4) | 43 (55.9) |
| Body mass index Kg/m^2^ (SD) †* | 23.9 (± 4.2) | 25.8 (± 4.7) |
| Years since onset (SD) † | 21.3 (± 9.0 ) | 27.7 (± 15.4) |

*Supplementary table:*

*† = significant difference between MS and HSP/HA patients (p > 0.05 x^2^ test)*

** = variable in question was associated with bone measures AND differed significantly between MS and HSP/HA patients, thus considered confounder and included in multivariate linear regression.*
